# Reminiscences of Buffalo General Hospital1Delivered before the Nurses’ Training School, March 11, 1904.

**Published:** 1904-04

**Authors:** Devillo W. Harrington

**Affiliations:** Buffalo, N. Y.


### Reminiscences of Buffalo General Hospital.[1]

By DEVILLO W. HARRINGTON, M. D., Buffalo, N. Y.

I HAVE been asked to say a few words this evening on the subject of hospitals in general and the Buffalo General Hospital in particular. The first authentic history of the founding of hospitals for the poor, was by the Buddhist priests in India, about 220 years before Christ. We have no record that seems authentic, of hospitals founded for the care of the sick and wounded in the early Jewish wars. Josephus in his history of that time makes no mention of hospitals.

The object of a hospital, is a place where patients may recover from sickness and injuries, in the shortest possible time with the smallest mortality and at least cost consistent with efficiency. Hospitals are in a way, a measure of the civilisation of the people. They are better adapted as to the nursing of patients, and better constructed and more numerous in proportion, as communities are more humane and more intelligent. As asylums for the sick and injured, they are the most splendid expressions

1. Delivered before the Nurses' Training School, March 11, 1904.

of wisdom and benevolence. So important is their proper and judicious management to the sufferer, that negligence in this respect may produce more injury than sanguinary battles.

Hospitals are to be considered from two points of view: first, to provide healthy and commodious homes and attendance, medical and surgical; and second, to provide schools of instruction for students in medicine and surgery. To Christianity solely, do we owe the institution of hospitals for the reception and care of the sick and injured. It was Christianity alone, which revealed the truth that all mankind are one and that human nature is the same in all.

Cesar states, that on the night preceding one of his battles, he ordered the sick and wounded to be sent to the nearest town. This precaution of removing the sick and wounded and collecting them in the towns where they could enjoy repose and first assistance, is the principle of the institution of military hospitals. Severus ordered the chariots to follow the army on the march for the conveyance of the sick and wounded. These are the first ambulances on record. As late as the sixteenth century, soldiers when seriously wounded were discharged with a small annuity, to find their way home as best they might, a practice founded on the economic principle which prevailed at that time—namely, that it cost more to cure a soldier than to levy a recruit.

The Hotel Dieu in Paris, is said to date from the end of the seventh century. The earliest record of hospitals in England, was about the end of the eleventh century. Hospitals were intended as a place of benefit to the poor, but in the earlier history of hospitals they often proved the reverse, on account of ignorance on the part of the administration of the true principles of health. This was largely due to the improper isolation and separation of contagious and infectious diseases. Another cause of mortality was improper ventilation. Formerly no arrangements were made for ventilation in hospitals, schools or churches. The want of fresh air aggravates all diseases, diminishes the power of resistance and increases the death-rate. The want of attention to the details of cleanliness has been a fertile source of hospital diseases. Faulty diet has also its influence on mortality, although its effects are not so easily traced.

Up to a comparatively recent date, hospital accommodations were confined to the large cities. The epoch of founding and building hospitals was the eighteenth century and that of building and founding of schools and libraries was the nineteenth century. Prior to these dates, estates and moneys were usually given to the church. This brought about the condition of things which existed during the reign of Henry VIII., prior to the Reformation, when the church owned or controlled about one-eighth of the property in England.

The Buffalo General Hospital was organized in 1855. The west wing of the present building (the first one built), was completed in 1858. The administration building was built in 1880 and the new building erected in 1897 and 1898. The first patient was a Mrs. Lawson, the widow of a clergyman. This does not seem so long ago when you realise that she still lives as an inmate of an old ladies' home in this city. My first entry into, and intimate knowledge of this hospital was in 1865, near the close of the civil war. Arrangements were made with the hospital authorities of the United States Government, for the admission of soldiers into this hospital, as well as into the Sisters of Charity Hospital, which was then located on Main, at the corner of Virginia street. These two hospitals were filled with sick and wounded soldiers, from, I think, sometime in 1864 until the close of the war.

In this old building were four wards. The wards were crowded with beds as close as they could be placed, and even the private rooms were filled with beds occupied by soldiers and the grounds of the hospital, east of the old building, were covered with tents filled with sick and wounded soldiers.

The medical officer in charge of this hospital, and also of the sick and wounded at Fort Porter and the Sisters' Hospital, was Major Crispell. He was a United States surgeon of wonderful executive ability and devoted his time to looking in detail after the care of each invalid soldier. The medical and surgical staff connected with the hospital was under the immediate charge of this surgeon. Drs. Miner and Eastman were the surgeons. Drs. Wyckoff, Rochester and Mixer were the attending physicians. Cases suffering from gangrene were occasionally sent here from the front. It was not uncommon for gangrene to develop in these overcrowded wards. It was only toward the close of the war that these patients were isolated and put in tents, or were removed to the second story of the barn which stood near where the contagious hospital is now located.

There were no trained nurses at that time. A Mrs. Peck had charge of the two wards upon the second floor, which were the surgical wards. A Mrs. Graham was in charge of the two lower wards which were medical. The two internes were Dr. J. N. Brown, who is now practising at Westfield, and Dr. E. L. Shurly, who is now in Detroit. The male nurse, or orderly, was a retired English soldier, by the name of MacDonald. He had lost an eye in the Crimean War and drew a pension from the English government. He had to go to Fort Erie to draw his pension and usually came back gloriously drunk. He had a way of getting into the laboratory through the window and getting drunk on the hospital tonic, which was made from the formula of Huxam's tincture of cinchona.

The two female nurses in the surgical wards were two sisters by the name of Anna and Jessie Gibson. They were without any training as nurses and yet did good work under the instruction of the internes. One died soon after the war and the other married a soldier, is now living in Mayville, and draws a pension as an army nurse. The superintendent of the hospital at that time was William C. Bagley, a most conscientious and capable man. His wife was matron. A Miss Sarah Hare was assistant matron.

Antiseptics in the way of surgical dressings were entirely unknown at that time. The first antiseptic dressing in this hospital was used upon a soldier brought from the front, who had lain three days upon the field after being wounded and seventeen days with no covering but the heavens. It was pronounced a hopeless case by the attending surgeons and permission was given to the internes to use any dressing for the wound that they thought proper. Permanganate of potash was the remedy that suggested itself to them and this was used most thoroughly and effectively. I have a very distinct recollection of the interest which the entire staff manifested in this new method, as I was the patient.

The first hypodermic injection in this city or in Western New York was used in this hospital. The first use of the thermometer and the keeping of records of the temperature of patients, was in this hospital. The first successful skin-grafting in this hospital, was in 1870 or 1871. Dr. Miner at a clinic before the surgical class, tried this upon a patient at the Sisters' Hospital, but without success. The interne here at that time (my innate modesty prevents my mentioning his name), tried it upon a patient in this hospital the same day, with perhaps more care as to detail, and it was a success.

Visitors by the thousands, mostly women, flocked into these wards at all hours of the day. There were two reasons for this: one was the attraction which brass buttons always have for the female sex, and the other, curiosity, which is the strongest instinct in women. I remember a soldier by the name of Darrow, who had a severe wound of the forearm. As the nurses were dressing this one day, a kind-hearted young woman said to him, "Soldier, is that the only place where you were hit?" He glared at her an instant and replied, "Madam, would you like to see a man shot all to pieces?" Another soldier by the name of Peterson, who was a blacksmith, was having his leg dressed for a compound comminuted fracture. One of these sympathetic women said to him, "Soldier, were you hit with a minnie or a bombshell." "Madam," said he, "I was kicked by an army mule." There was no further interest manifested in his case. Another soldier who was around the ward on crutches was the center of an admiring group of sympathetic young women, who were asking him about the battle in which he was wounded, if the bullets fell around him, and if the shells were bursting in the air, and finally one of them asked him how he felt at this time. He said he felt as if things were coming his way.

A thousand memories; both humorous and pathetic, come back to me like a panorama and as vividly as if it were yesterday, when I think of the muster out and final home-going, during the closing days of the civil war.

"The story of the muster-out roll is, at best, but a sad one. We are carried back to the war and surrounded by its sad picture."

" 'Killed, July 3, at Gettysburg;' and one thinks of Pickett's charge, or other incidents of that historic field."

" 'Killed, May 3, 1863, at Marye's Heights;' and the compiler lays down his pen to dream again of that fierce charge which swept upward over the sloping fields of Fredericksburg."

" 'Wounded and missing, May 6, 1864, at the Wilderness;' suggests a nameless grave, marked if at all, by a government headstone bearing the short, sad epitaph, 'Unknown.' "

" 'Killed at Malvern Hill, July 1, 1862;' and there rises a picture of an artilleryman lying dead at the wheels of his gun."

" 'Died of gunshot wounds before Atlanta, August 20, 1864;' tells of some lad who fills a grave long miles away from the village churchyard of his Northern home."

" 'Wounded at Antietam, September 17, 1862, and died on the amputating table;' brings up the dire vision of the field hospital, that ghastly sequel to every battle."

" 'Killed at Appomattox, April 9, 1865;' and one sees the dead cavalryman, who, falling in that closing battle of the war, died with home and victory in sight."

" 'Died of sunstroke,' recalls the long march, the heavy road, the dust, the heat, and a senseless form lying at the roadside."

" 'Killed on picket, September 15, 1863, on the Rappahannock;' suggests the star-lit river, the lonely vidette, an echoing shot, and a man dying alone in the darkness."

"And so it goes. There were no war stories that can equal the story of the muster-out roll. War is terrible, but the sequel is horrible."

The hospital grounds were enclosed at this time by a high picket fence. The brick guard-house which was torn down last spring, stood at the only entrance to the hospital grounds. An English soldier who had served in India and was at the Sepoy mutiny, was constantly on guard and no patient was allowed to leave the grounds, without a pass issued from the office. A large lot bounded by the hospital grounds, High, Ellicott and Goodrich streets, was not built upon and was used by the hospital as a garden. This continued, I think, until about 1875. All the water that was used in and around the hospital was drawn in a water-cart from the corner of Main and Virginia streets. There was a large cistern just east of the old building, into which these water-carts were emptied and the water was pumped from there through the building. The wards were heated entirely by large coal stoves.

My next entry into this hospital was as interne in the fall of 1869. There were no trained nurses during the two years and nine months in which I served. There was the same staff of medical and surgical attendants as in 1865, with the addition of Dr. Gay upon the surgical staff and Dr. Brown upon the medical staff. The internes at this time were designated as resident physician and assistant resident physician. Dr. L. H. Kitchell was my predecessor, and I served under him one year as his assistant. He was a graduate of Yale College and one of the brightest students ever graduated from the medical department of Buffalo University. He was energetic and thorough and to him I owe my earliest training in the practice of medicine.

When I became resident physician, after serving one year as assistant, Dr. P. W. Van Peyma was appointed my assistant. He was careful, capable and conscientious, traits which have characterised him all these years in his practice in this city. There was no pavement at this time on High street, and for several months during the year this street was almost impassable. The hospital had no ambulance during my service here; in fact, there was not an ambulance in this city. Patients were brought to and from the hospital in carriages, or in a wagon which the hospital owned, which I think had been bought second-handed from some grocer.

Upon the resident physician and his assistant, devolved all the work, including most of the dressings in and around the hospital, keeping records, putting up medicines in the laboratory and attending obstetric cases, of which we had a large number at that time. The resident physician and his assistant usually made their rounds together, as the service was of mutual benefit to both. When I first entered the hospital as assistant resident physician, it was customary for the patients who were not confined to their beds, to sit around the stoves or remain in the washroom, during the rounds of the residents or the visiting staff. After a few days of this seeming carelessness, I suggested to my superior, Dr. Kitchell, and the superintendent, Mr. Bagley, that each and every patient be in or at his bed during the rounds of the residents or any member of the visiting staff. This was a marked innovation and the results were so satisfactory, that it has been carried out to the present time.

The consulting staff was Drs. Burwell, Strong, White, Loomis and Hauenstein. Most of the nursing at this time was done by those who had been patients in the hospital and during their convalescence had picked up a smattering of the duties of nurse or orderly. A detail was made from the convalescents to work in the garden, mow the lawn and keep the grounds clean around the hospital.

The Sisters of Charity Hospital and General Hospital alternated each year in the care of the marines or sailors from this port. This increased largely the number of patients each alternating year, and they were the worst class of patients that we had to manage. Card playing was prohibited at this time, as it was also when the hospital was filled with soldier patients. Soldiers accustomed to the liberty and license of three years or more of field service, objected seriously to this order. I remember a soldier by the name of John Sabin, the son of a Baptist minister and as brave a soldier as ever drew saber, who persistently violated this rule. For discipline, he was sent to Fort Porter, and, I think, kept upon bread and water for ten days. This he said was a worse punishment than his father had ever given him for a similar offense. He is now a prominent physician in a Western city. Cigarette smoking was prohibited and was one of the bad habits to which the internes were not addicted. I regret that I cannot say as much for the internes at present.

I would be remiss in this discourse did I neglect to mention the name of Charles E. Clark, one of the founders of this hospital and president of the board of trustees for many years. More, perhaps, is due to him than to any other man for the founding of this hospital and its successful management during its early days. I remember how anxious he was for the hospital to purchase the lot between the hospital grounds and Ellicott street, of which he was the owner or agent, and which could have been bought at that time for $5,000.

Two hundred and seventy-one nurses have been graduated from the training school, which was founded in 1877. One of the beautiful spots in Washington, is Dupont Circle, in the center of which stands a magnificent statue of Admiral Dupont, one of the old-time gallant officers of our navy. It is said of him that on his death bed he gave this toast: "Here's to my country, may she ever be right, but right or wrong, my country." May each and every one of you nurses and internes, ever manifest the same spirit of loyalty to the Buffalo General Hospital.

1430 Main Street.

